# Lymphoepithelioma-Like Carcinoma of the Breast: A Case Report Unveiling Several Clinical and Histopathological Challenges

**DOI:** 10.1155/2018/8240534

**Published:** 2018-07-12

**Authors:** Tarek Aridi, Mohamad Fawwaz, Ahmad Kassab, Marwan Bahmad, Faisal Houcheimi, Mohamad Mshiek, Fouad Boulos, Ali Kanj, Ghassan Ramadan, Hisham F. Bahmad, Najla Fakhruddin

**Affiliations:** ^1^Faculty of Medicine, American University of Beirut, Beirut, Lebanon; ^2^Faculty of Medicine, Beirut Arab University, Beirut, Lebanon; ^3^Department of General Surgery, Hammoud Hospital University Medical Center, Saida, Lebanon; ^4^Department of Pathology and Laboratory Medicine, American University of Beirut Medical Center, Beirut, Lebanon; ^5^Department of Radiology, Hammoud Hospital University Medical Center, Saida, Lebanon; ^6^Department of Anatomy, Cell Biology and Physiological Sciences, Faculty of Medicine, American University of Beirut, Beirut, Lebanon; ^7^Department of Pathology, Hammoud Hospital University Medical Center, Saida, Lebanon

## Abstract

Lymphoepithelioma-like carcinoma (LELC) of the breast is an extremely rare tumor type. Histologically, it mimics undifferentiated nasopharyngeal carcinoma by demonstrating nests of neoplastic epithelial cells in a background of lymphoplasmacytic infiltrates. This paper reports a 62-year-old female patient with a 3 × 1.5 cm BI-RADS type IV breast mass diagnosed on excisional biopsy as LELC. The tumor is negative for estrogen and progesterone receptors and did not overexpress HER2/neu. Routine tests for clearance before surgery were performed, and patient was managed by a modified radical mastectomy with axillary lymph node dissection showing no residual tumor. Surgical CAse REports (SCARE) guidelines were followed for reporting our case. The rarity of LELC of the breast warrants the establishment and implementation of well-defined guidelines and criteria for diagnosis and management.

## 1. Introduction

Lymphoepithelioma-like carcinoma (LELC) of the breast is an extremely rare malignancy with 32 cases reported in the world literature to date [1]. In 1994, Kumar and Kumar described the first case of LELC in the breast of a 65-year-old woman, in which utilizing immunostaining of the tumor sections revealed scanty epithelial neoplastic cells with copious lymphocytic infiltrates [2]. Morphologically, the tumor resembled nasopharyngeal lymphoepithelioma (old terminology for undifferentiated nasopharyngeal carcinoma) and other similar tumors occurring in different organs such as the stomach, salivary glands, lungs, thyroid, and uterus [3].

Histologically, LELC of the breast is characterized by ill-defined cohesive nests of malignant epithelial cells within a background of dense and diffuse lymphoid infiltration that is intimately mixed with the tumor [1, 3]. Although Epstein-Barr virus (EBV) has been linked to the pathogenesis of LELC of the nasopharynx, salivary glands, stomach, and others, it has never been associated with breast LELCs [1, 4].

**Figure 1:**
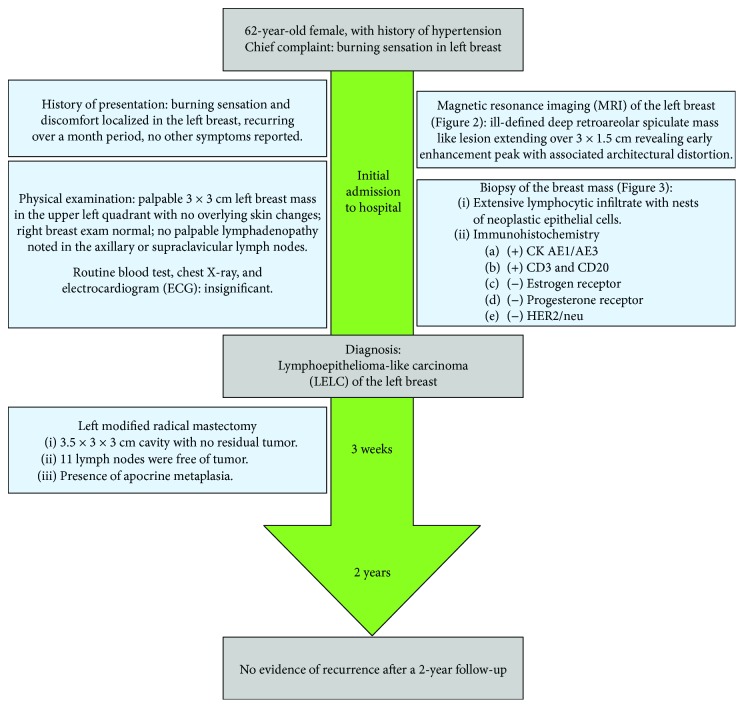
Timeline organizing main events of the case.

In this paper, we present a case of LELC of the breast, with a minireview of the literature. We also describe the differential diagnoses and the therapeutic approaches that have been adopted in the treatment of this tumor. This surgical case report (Figure 1) was conducted and reported in accordance with Surgical CAse REports (SCARE) guidelines for reporting case reports.

## 2. Case Presentation

A 62-year-old female patient who is heavy smoker presented with a burning sensation and discomfort in her left breast that has been recurring over a month prior to admission to the hospital. No fever, chills, or any other symptoms were described. She reported a past medical history of hypertension and a surgical history of hemorrhoidectomy, dilation and curettage surgery, colonoscopy, and gastroscopy.

Physical examination revealed a palpable left breast mass (measuring approximately 3 × 3 cm) in the upper quadrant with no overlying skin changes. The right breast exam was normal. No palpable locoregional lymphadenopathy (axilla and supraclavicular lymph nodes) was noticed. Routine blood tests (complete blood count with differential, electrolytes, prothrombin time, partial prothrombin time, and international normalized ratio), chest X-ray, and electrocardiogram (ECG) were all normal.

**Figure 2:**
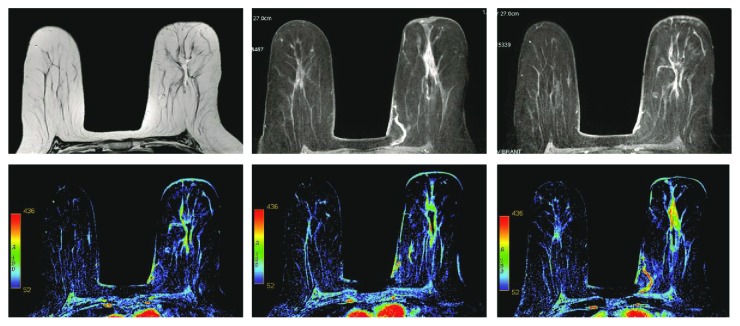
Breast MRI. Ill-defined deep retroareolar spiculate mass-like lesion extending over 3 × 1.5 cm revealing early enhancement peak with associated architectural distortion.

Magnetic resonance imaging (MRI) of the left breast showed an ill-defined deep retroareolar spiculate lesion extending over 3 × 1.5 cm revealing early enhancement peak with associated architectural distortion. There were no axillary lymph nodes or abnormal bone signal intensity. No cutaneous thickening or retraction was seen. Findings were suggestive of BIRADS type IV lesion (Figure 2).

**Figure 3:**
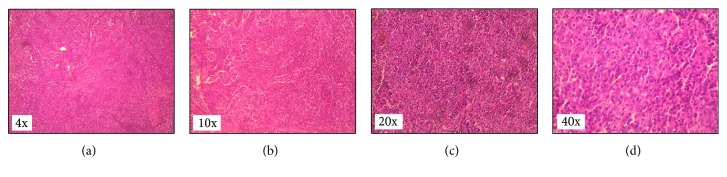
(a and b) Low magnification histopathological examination of the breast tumor demonstrating nests of neoplastic epithelial cells in a background of dense lymphocytic infiltrate. (c and d) Higher magnification showing the tumor cells with abundant pale cytoplasm, large vesicular nuclei, and prominent nucleolus (hematoxylin and eosin stain).

**Figure 4:**
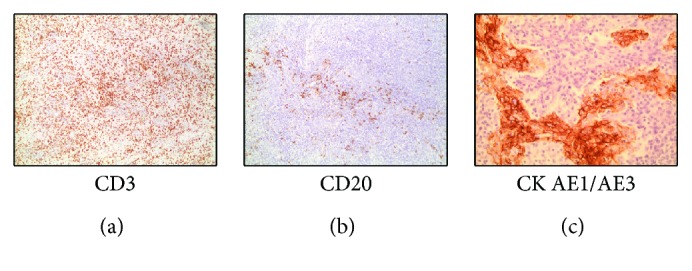
The lymphocytes show positive immunohistochemical staining for CD3 (a), CD20 (b), and the tumor cell-positive immunohistochemical staining for cytokeratin AE1/AE3 (c).

An excisional biopsy was performed and revealed breast tissue with extensive lymphocytic infiltrate intermixed with neoplastic epithelial cells (Figure 3). Immunohistochemistry results were positive for CK AE1/E3 antibody in the neoplastic epithelial cells with no expression of estrogen or progesterone receptors, and HER2/neu was not overexpressed (Figure 4(c)). The lymphocytes in the background stained positive for both CD3 and CD20 (Figures 4(a) and 4(b)).

The patient underwent a left modified radical mastectomy. Eleven lymph nodes were dissected and free of tumor. The mastectomy specimen showed a 3.5 × 3 × 3 cm cavity at the site of the previous excisional biopsy. On histological examination, apocrine metaplasia was identified but no residual tumor was detected. To note, apocrine metaplasia is a very common incidental benign finding that is considered part of or associated with fibrocystic changes, and hence, does not affect prognosis and management [5]. Accordingly, no adjuvant hormonal therapy, chemotherapy, or radiotherapy was given to the patient.

No evidence of recurrence was noted on a 2-year follow-up.

## 3. Discussion

Lymphoepithelioma-like carcinomas (LELCs) are a type of malignant tumors that can be found in any organ with an epithelial tissue origin such as the lungs, urinary bladder, thymus, colon, skin, prostate, and breast [6]. Microscopically, they mimic undifferentiated nasopharyngeal carcinoma which is known to be strongly associated with Epstein-Barr virus (EBV) infection. Similarly, LELCs of the thymus, salivary glands, lungs, and stomach are associated with EBV infection as demonstrated by Iezzoni et al. [4]. To our knowledge, none of the reported LELCs of the breast were associated with EBV infection (Table 1). Human papilloma virus (HPV) has been detected in two LELCs of the breast [3, 7], but Herrera-Goepfert et al. considered that the HPV viral load in breast neoplasms proved to be really low thus excluding its involvement in the carcinogenesis [8]. Therefore, based on current evidence, the etiology of LELC of the breast cannot be correlated with viral infection.

LELC of the breast is a rare histopathologic variant of breast carcinoma and is not a part of the World Health Organization (WHO) classification for breast cancer [9]. To the best of our knowledge, only 33 cases were published in the English literature until drafting this case.

The average patient age is 52 years (range: 37 to 69 years). The tumors have an average diameter of 2.5 cm (range: 1 to 4 cm). Metastasis to lymph nodes was observed in 29% of the cases (8 out of 28 examined cases), a finding consistent with their relatively favorable prognosis. Estrogen receptors were expressed in 28% of the cases while progesterone receptors were expressed in 13% of the cases. Majority of the cases with lymph node involvement (7 out of 8 cases) were negative for estrogen and progesterone receptors, while 16% (5 out of 31 cases) overexpressed HER2/neu (Table 1).

Although in most cases of breast lesions, mammography is usually the primary imaging modality used, recent studies have been emphasizing on the efficacy of MRI over mammography in detecting and assessing such breast lesions. In one study by Roganovic et al., it has been shown that “sensitivity for digital mammography, breast MRI, and breast tomosynthesis was 72.4%, 93.1%, and 100%, respectively, while the specificity was 46.4%, 60.7%, and 75%, respectively” [10].

In order to avoid misdiagnosis of LELC of the breast, it is important to consider neoplasms of the breast with evident lymphocytic infiltrate, such as lymphomas and medullary carcinomas, in the differential diagnosis. A reported case of LELC was originally misdiagnosed as lymphoma or pseudolymphoma as a result of a dense lymphocytic component concealing the elusive neoplastic cells [11]. Another case of LELC of the breast mimicked sclerosing Hodgkin's lymphoma with a tumor section comprising nodular clusters of lymphocytic cells interspersed around a network of fibrous septae [12]. This necessitates the use of a panel of immunohistochemical markers with cytokeratin and leukocyte common antigen, to differentiate between lymphomas and lymphoepithelioma-like carcinomas [13]. LELC of the breast and medullary carcinomas share the presence of syncytial growth patterns, a dense lymphocytic infiltrate, and the absence of estrogen receptors [11, 14]. However, they vary grossly, with the medullary carcinoma being well circumscribed and demarcated as compared to LELC of the breast, and microscopically, with the medullary carcinomas' syncytial growth patterns covering an area greater than 75% [15]. Moreover, the lymphocytic component in medullary carcinoma does not obscure the epithelial component as extensively as it does in LELC of the breast [16]. Immunohistochemically, medullary carcinomas are usually E-cadherin positive, while LELC of the breast are negative [11].

Currently, there is no standard protocol for the treatment of LELC of the breast, due to the rarity of this tumor; nevertheless, several methods have been adopted. Radical or partial mastectomy is the most common practiced surgical procedure in all reported cases of LELC of the breast. In our case, modified radical mastectomy of the left breast with axillary lymph node dissection was performed. Moreover, many of the reported cases have received adjuvant chemotherapy and radiotherapy, while hormonal therapy was used for patients with positive estrogen or progesterone receptors.

Although there are a limited number of cases to evaluate the prognosis of LELCs of the breast, it is important to note that after years of follow-up, the majority (27 out of 33) had no evidence of recurrence or metastasis postsurgery and therapy. Therefore, LELC of the breast can be considered to have a favorable prognosis. No molecular characterization of LELC of the breast has been reported yet. Therefore, further studies are needed to better characterize this tumor and may provide an optimal therapeutic protocol for LELCs of the breast in the future. In addition, it is strongly recommended to include LELC as a part of the WHO classification for breast cancer, which may consequently aid in correlating clinicopathological findings associated with LELC with the classic histopathological classification of breast cancer.

## 4. Concluding Remarks

Lymphoepithelioma-like carcinoma of the breast is an extremely rare entity with a favorable outcome when treated; therefore, it should be considered when diagnosing breast tumors with a rich lymphocytic infiltrate. Sometimes LELC of the breast maybe misdiagnosed as lymphoma or as medullary carcinomas. Accurate diagnosis can be attained with good evaluation of the morphology and correlation with immunohistochemistry findings. We expect to see more cases of LELC of the breast reported in the literature; therefore, well-defined guidelines and criteria for diagnosis and management of an LELC in the breast are warranted.

## Figures and Tables

**Table 1:** Table summarizing main clinicopathological parameters of LELC cases reported so far in world literature.

Number	Authors and reference	Year	Patient age (years)	Tumor size (cm)	Lymph node	ER	PR	Her2	CK AE1/AE3	EBV
1	Kumar and Kumar [2]	1994	65	2.0	0	+	+	−	+	−
2	Cristina et al. [17]	2000	54	1.5	0/19	+ (42%)	− (<10%)	−	NA	−
3	Dadmanesh et al. [14]	2001	43	1.9	1/1	−	−	−	NA	−
4			53	2.0		−	−	−	NA	−
5			49	1.0	0/19	−	−	−	NA	−
6			52	2.7	0/20	+	−	−	NA	−
7			64	2.0	0/29	−	−	−	NA	−
8			69	2.3	0/19	−	−	−	NA	−
9	Naidoo and Chetty [18]	2001	50	2.5	2/24	NA	NA	NA	−	−
10	Pestereli et al. [19]	2002	56	2.0	2/27	+	+	−	+	−
11	Sanati et al. [11]	2004	62	3.0	NA	+ (10%)	−	−	+	−
12	Ilvan et al. [16]	2004	59	3.5	0/20	+	+	−	+	−
13			67	1.1	0/16	−	−	−	+	−
14	Kurose et al. [20]	2005	47	2.8	0/33	−	−	+	+	−
15	Saleh et al. [12]	2005	51	1.3	1/8	−	−	NA	+	−
16	Kulka et al. [7]	2008	42	2.5	0/10	+	−	−	+	−
17	O'Sullivan-Mejia et al. [21]	2009	55	3.1	0/2	−	−	+	+	−
18	Jeong et al. [22]	2010	37	3.0	0/13	−	−	+	+	−
19	Dinniwell et al. [6]	2012	55	4.0	0/2	−	−	−	NA	−
20	Nio et al. [3]	2012	45	3.0	0/5	−	−	−	NA	NA
21	Suzuki et al. [23]	2012	64	2.1	3/23	−	−	+	NA	NA
22	Trihia et al. [24]	2012	53	1.5	2/30	−	−	+	+	NA
23	Abdou and Asaad [13]	2014	45	2.0	0/24	−	−	−	NA	−
24	Top et al. [25]	2014	59	3.0	0/23	−	−	−	NA	−
25	Nankin et al. [26]	2015	39	2.7	0/5	+ (40%)	−	−	NA	NA
26	Sato et al. [27]	2016	50	1.2	1/23	−	−	−	+	−
27	Herrera-Goepfert et al. [28]	2016	57	4.0	0	+	+	−	+	−
28	Shet et al. [1]	2016	56	3.0	1/17	−	−	−	NA	−
29			39	2.0	0/18	−	−	−	NA	−
30			40	2.5	NA	−	−	−	NA	−
31			40	3.5	NA	−	−	−	NA	−
32			51	3.0	NA	−	−	−	NA	−
33	Present case	2017	62	3.5	0/11	−	−	−	+	NA

ER: estrogen receptor; PR: progesterone receptor; Her2: Her2 receptor; CK: cytokeratin; EBV: Epstein-Barr virus; NA: not available.

## Abbreviations

BI-RADS: Breast imaging reporting and data system
EBV: Epstein-Barr virus
ECG: Electrocardiogram
HPV: Human papilloma virus
LELC: Lymphoepithelioma-like carcinoma
MRI: Magnetic resonance imaging
SCARE: Surgical CAse REports
WHO: World Health Organization.
